# Diphtheria

**Published:** 1884-02

**Authors:** E. B. Nash

**Affiliations:** Cortland, N. Y.


### DIPHTHERIA.

E. B. Nash, M. D., Cortland, N. Y.

In the *Hahnemannian Monthly*, for September, there is an article by Dr. C. Neidhard on the treatment of this dread disease. Boiled down, it amounts to this—Liquor Calcis Chlorinatæ is *the* homœopathic *specific* for diphtheria from a non-symptomalogical standpoint.

Now, a long time ago, when Dr. Burt made a proving of Phytolacca dec. he predicted from that proving that Phyto. would be a good remedy for diphtheria. That prediction has been abundantly verified. Jahr wrote: "Since I have become acquainted with Apis, I prefer this to any other remedy, and accomplish my purpose with it better than with any other medicine. Dr. Fetterhoff, myself, and many others have in practice witnessed its wonderful efficacy."

Dr. Boskowitz, of Brooklyn, lauded the virtues of Ignatia, and Wm. C. J. Slough, during an epidemic in Lehigh County, Pa., did not lose a single case after he commenced using Ignatia$^{200}$.

Dr. Reissig, of New York, used Lac. caninum with great success. Dr. Dunham got the secret, while Dr. Swan has *proved* the remedy and done more to perfect our knowledge of this agent than any other man.

Dr. Beck, and after him Von Villers, from actual experience laud Merc. cyanuret as a great specific in diphtheria.

Now, without making particular mention of Lach., Lyc., Merc. prot., Merc. biniod, Kali bich., and some others which have performed no very mean service in the treatment of this disease, one very important question forces itself upon us—which is the best? Taken from a pathological or symptomalogical standpoint, the *results, ab usu in morbis*, must after all, it seems to me, decide as to the merits of the remedy.

Testimony seems to be about equally favorable for each. Is it, then, a fact that it makes no difference which we use?

It seems to me that the only logical conclusion is—that each and all of these remedies have proved themselves very valuable. Second, that there must be some rule by which we may decide in favor of one or the other in particular cases. It is so in other diseases. Why not in this? How would it do to lump this business for typhoid fever, scarlatina, rheumatism, and all the long list of diseases that flesh is heir to, in the same way that Dr. Neidhard does for diphtheria? Baer tried to do that for typhoid by stating that Arsenic ought to be given early in the disease as well as late, because in its *general character* it most resembled the general character of typhoid. If diseases always pursued the same and an unvarying course, and had no modifying circumstances as to causes, climate, temperament, complications, etc., this method might do, but as long as these are present and must be, it is not and cannot be scientific, or attended with the best results possible. Dr. N. says: "It has been remarked by others, as well as myself, that the selections of a remedy from *unimportant symptoms* will not lead to the true simillimum in any important case," and says it will often lead us astray. This is very true, and the only question that remains is, What are the important and what the unimportant symptoms? If a man would select his remedy on unimportant symptoms alone, of course he would be led astray, and I think he will be led almost equally astray if he undertakes to confine himself to one remedy for all cases selected from the "similarity of the general character of the remedy to the character of the disease."

If this is not so, won't Dr. N. please make some more "thorough and exhaustive studies," and give us the one remedy *par excellence* for all diseases. If he can do it for diphtheria, we doubt not his ability to do it for some of the rest. How much time and labor, and often failure at that, it would save the profession in general. Now I do not doubt Dr. N.'s testimony in favor of his remedy for diphtheria in so far as that he has been successful with it in many cases, but his testimony is no more entitled to our credence than that of those who have found other remedies equally efficacious. On the other hand, I cannot believe that the case to which chloride of lime is homœopathic could be equally well cured by Merc. cyanuret, or *vice versa*. In my mind the case comes to just this: There is no specific for any disease in all its forms, and that although, as taught by Hahnemann, Hering, and others, we may sometimes find a remedy that corresponds well to the *genus epidemicus*, and find it useful in the majority of cases; yet, as taught by the same men, each case should be treated as though we had never had one like it before—that is, the case must be *covered* by the remedy so far as is possible in both its pathological and *symptomalogical entirety*. Unimportant symptoms! any symptom actually belonging to either drug-proving or disease, cannot in the nature of things be unimportant. How would it do in a case of brain disease to ignore the delirium of Stram. or Bell., the sopor of Opium, or the screams of Apis, and prescribe for the pathological condition? Now, lest I be misunderstood, I am not charging upon Dr. N. such practice in general—I have greater faith in him. But when a man asks me what I give for diphtheria shall I say "Chloride of lime"? Would it not be more in keeping with the spirit of Homœopathy to say, "I give the *indicated remedy* for each case by itself"?

